# Synaptic Response of Fluidic Nanopores: The Connection of Potentiation with Hysteresis

**DOI:** 10.1002/cphc.202400265

**Published:** 2024-10-04

**Authors:** Juan Bisquert, Marc Sánchez‐Mateu, Agustín Bou, Cheryl Suwen Law, Abel Santos

**Affiliations:** ^1^ Instituto de Tecnología Química Universitat Politècnica de València-Agencia Estatal Consejo Superior de Investigaciones Científicas) Av. dels Tarongers 46022 València Spain; ^2^ Institute of Advanced Materials (INAM) Universitat Jaume I 12006 Castelló Spain; ^3^ Leibniz-Institute for Solid State and Materials Research Dresden Helmholtzstraße 20 01069 Dresden Germany; ^4^ School of Chemical Engineering The University of Adelaide Adelaide, South Australia 5005 Australia; ^5^ Institute for Photonics and Advanced Sensing The University of Adelaide Adelaide, South Australia 5005 Australia

## Abstract

Iontronic fluidic ionic/electronic components are emerging as promising elements for artificial brain‐like computation systems. Nanopore ionic rectifiers can be operated as a synapse element, exhibiting conductance modulation in response to a train of voltage impulses, thus producing programmable resistive states. We propose a model that replicates hysteresis, rectification, and time domain response properties, based on conductance modulation between two conducting modes and a relaxation time of the state variable. We show that the kinetic effects observed in hysteresis loops govern the potentiation phenomena related to conductivity modulation. To illustrate the efficacy of the model, we apply it to replicate rectification, hysteresis and conductance modulation of two different experimental systems: a polymer membrane with conical pores, and a blind‐hole nanoporous anodic alumina membrane with a barrier oxide layer. We show that the time transient analysis of the model develops the observed potentiation and depression phenomena of the synaptic properties.

## Graphical abstract

The performance of fluidic networks for brain‐like computation applications depends on short term memory properties of the rectifying elements. We show the connection of synaptical property of nanofluidic pores to the hysteresis behaviour. Potentiation and depression are connected to intrinsic inductive and capacitive behaviours caused by the impeded ion conduction mechanism.

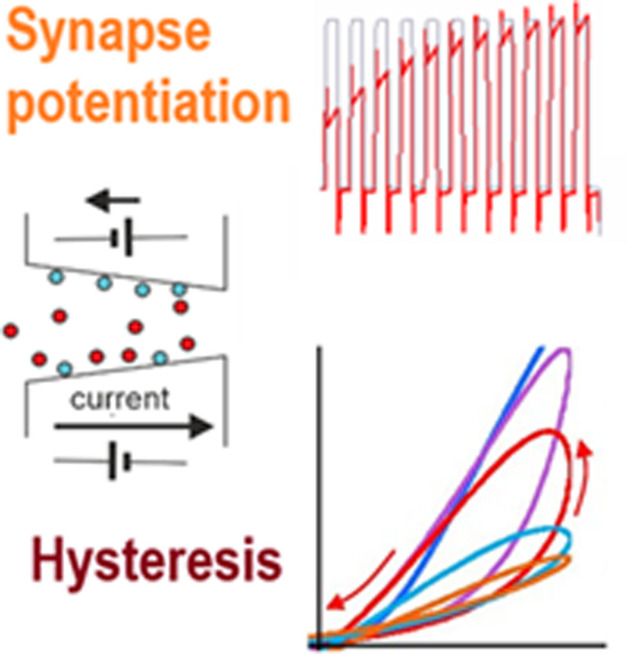

## 1 Introduction

Neuromorphic computing systems, engineered to emulate the intricate processes of biological neural networks, hold immense promise in transcending the constraints inherent in conventional AI algorithms and classical computing paradigms. By leveraging the parallelism, fault tolerance, and energy efficiency characteristic of the human brain, these innovative systems offer a transformative approach to computing.[^1^, ^2^, ^3^, ^4^]

Biomimetic material devices inspired by these biological structures offer the capability to replicate various neuromorphic functions, including artificial synapses, neurons, and dendrites. Ionic current rectification is a commonly observed phenomenon in both naturally occurring protein ion channels and synthetic nanopores.^[5]^ Artificial solid‐state nanopores and nanochannels can serve as synthetic counterparts to protein ion channels and have been tailored for various sensing applications,[^6^, ^7^, ^8^, ^9^] micro‐nano pumping devices^[10]^ and as artificial neurons.[^11^, ^12^, ^13^] Diode properties of nanopores have been researched widely.[^6^, ^14^, ^15^, ^16^, ^17^, ^18^, ^19^, ^20^, ^21^, ^22^, ^23^]

In the brain, synapses are the elements that connect neurons and determine the flow of information, by adjusting the response they receive form the neighboring neurons.^[24]^ Natural synapses either reinforce or weaken by repeated concerted stimuli, according to the strength and synchronization of the perturbations. To realize these properties in a material computational network, the synaptic weight of the device must adapt by increasing the conductivity to successive input stimuli, and the gain must be persistent by the time used in computation. Synaptic devices must therefore possess distinct resistive states that correspond to controlled synaptic weights.[^25^, ^26^, ^27^, ^28^, ^29^]

A wide array of material platforms, have been tested for the development of synapses.[^4^, ^30^, ^31^] It has been shown that fluidic and artificial solid‐state nanopores can be applied in computation networks composed of neurons and synapses.[^11^, ^12^, ^13^, ^24^, ^32^, ^33^, ^34^, ^35^, ^36^, ^37^, ^38^, ^39^, ^40^, ^41^, ^42^, ^43^] Recently, the synaptic properties of polymer membranes with conical nanopores have been reported.[^43^, ^44^, ^45^, ^46^] However, the kinetic response of these devices and how they cause synaptic properties is still poorly understood.

Fluidic nanopores are also candidates for applications in physical reservoir computing systems, in which dynamic memristors project the features of the temporal inputs into a high‐dimensional feature space.[^47^, ^48^] The short‐term memory (fading memory) is a dominant property for this type of computation.^[49]^

In this article, we present a general model which can reproduce systematically the synaptic properties of multipore membranes with rectifying nanofluidic pores. The model^[46]^ is inspired on general approaches of the Hodgkin and Huxley (HH) model[^50^, ^51^, ^52^, ^53^, ^54^] that describes the dynamics of biological neurons, and in memristor models.[^6^, ^14^, ^15^, ^16^, ^17^, ^18^, ^19^, ^20^, ^21^, ^22^, ^23^] These operational features are gauged by established measurements replicated from biology, such as the paired‐pulse facilitation (PPF) – a significant short‐term phenomenon that has been extensively examined in the field of neuroscience.^[55]^ Judiciously designed positive and negative input voltage pulses are applied to the model to analyze the transient response. We show that the kinetic effects that are easily observed in hysteresis loops are also responsible for the potentiation phenomena related to conductivity modulation, as previously reported in perovskite memristors.[^56^, ^57^] We emphasize the significance of the relaxation time that control the long time (inductive) current increase response.

## 2 Rectification and Hysteresis

**Figure 1:**
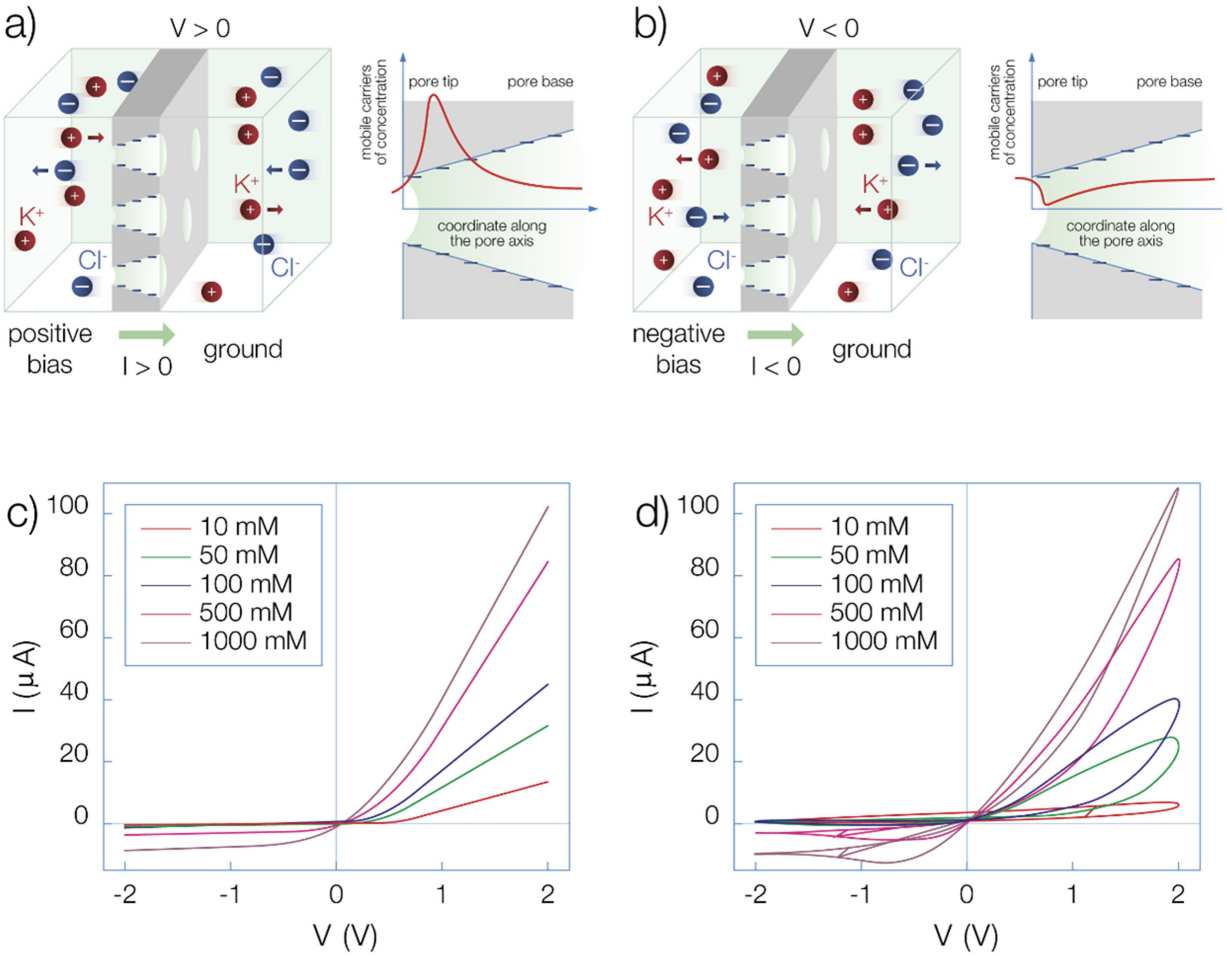
(a and b) Schematic representation of the mechanism of rectification that is obtained when the voltage applied to the cell is inverted in polarity giving higher (a) or lower (b) current according to the dominant charge accumulation in the tip of the pore with negatively charged walls. (c and d) Current‐voltage curves of the nanoporous sample with different concentrations of KCl. Experimental curves at quasi‐steady state (c) and at 10 Hz of frequency (fast scan), displaying hysteresis (d).

The nanoporous polymer membranes with synaptic response^[43]^ have been obtained by irradiation of 12.5 μm thick polyimide (PI) foils with swift heavy ions at UNILAC linear accelerator (GSI, Darmstadt). The passage of the ions through the polymer film leads to the formation of latent ion tracks that are converted into asymmetric, approximately conical pores using the track‐etching procedure, Figure 1.[^58^, ^59^] The pores show diameters around 20–50 nm (tip) and 400–800 nm (base). The number of pores in the sample can be controlled by adjusting the fluence of the impinging ions (ca 300 pores in our case). The track‐etching procedure results in the formation of carboxylate groups that give negative charge densities on the pore surface.^[60]^ Measurements were made with a BioLogic SPL‐200 potentiostat. Stationary I−V curves were measured with triangular waves. The scan rate was 200 mV/s. Loop curves were measured with sine waves of 10 Hz frequency. When the membrane is placed in an electrochemical cell separating two KCl solutions, it shows a characteristic rectification behavior, with low resistance for the voltage V>0 , when the counter ions (K^+^) are driven by the electric current into the pore tip (Figure 1a), and high resistance for V<0 currents, when the counter ions are driven out of the pore tip (Figure 1b). The dependence on geometric factors and surface charges has been explained elsewhere.[^20^, ^43^] The current asymmetries arise from the different ionic concentrations available for conduction and the distinct Debye screening of the negative pore tip charges. The extent of rectification is modulated by the electrolyte concentration as shown in Figure 1c and d.

Hysteresis is the effect in which the current depends on the direction and velocity of the voltage variation. In the electrochemical technique of cyclic voltammetry the voltage u is swept in the range $\left({-u}_{1},u_{1}\right)$ at a constant scan rate $v_{s}$ as

(1) $$u=v_{s}t\;$$

Alternatively the voltage can be swept at a constant angular frequency $\Omega_{s}$ as follows:

(2) $$u=u_{1}sin\left(\Omega_{s}t\right)\;$$

Comparing a full cycle in both systems, the following relationship holds between the velocity and the frequency:

(3) $$\Omega_{s}=\frac{\pi v_{s}}{2u_{1}}\;$$

The general theory of hysteresis recently derived^[61]^ identifies two types of hysteresis: capacitive (clockwise loops) and inductive (counterclockwise loops). In Figure 1d the hysteresis properties are obtained consisting of an opening of the current–voltage curves measured at 10 Hz, in comparison with the quasi‐steady state measurement of Figure 1c. Additional characterizations of hysteresis in this system are presented in recent publications[^43^, ^44^, ^45^] and are discussed later in this paper.

**Figure 2:**
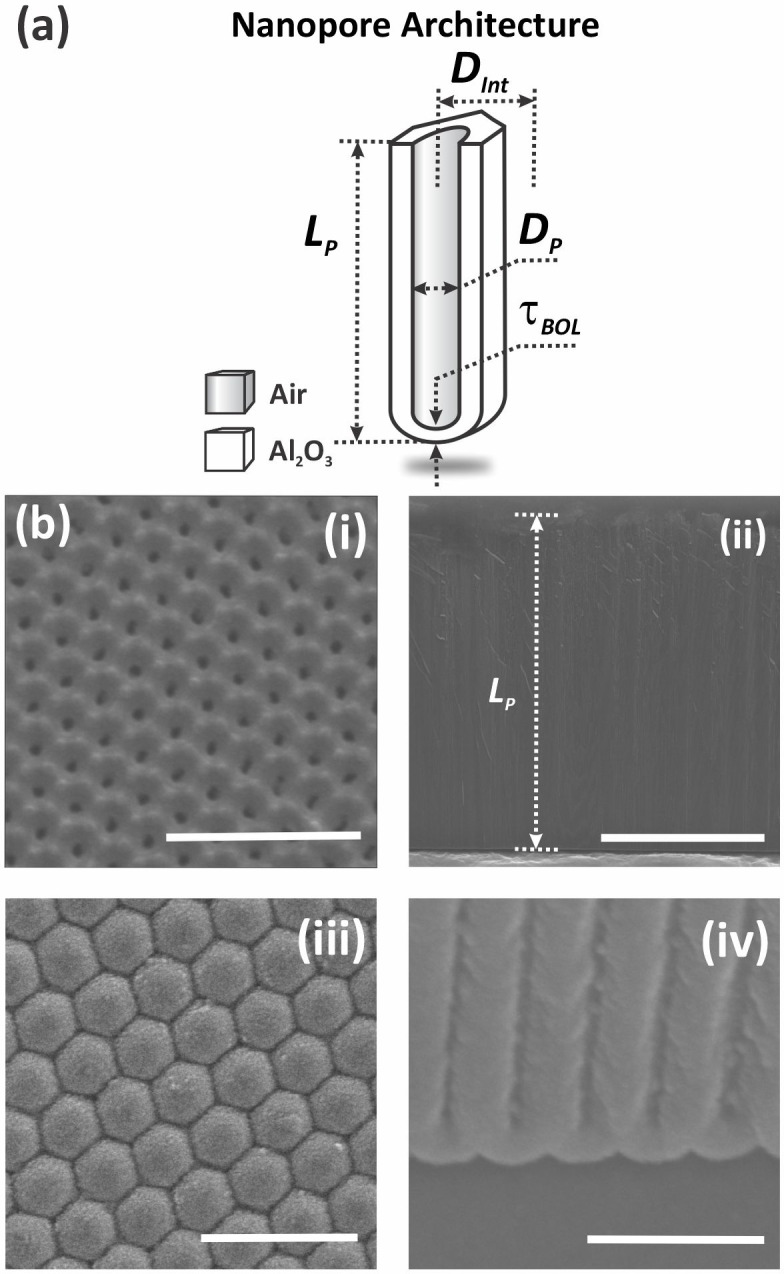
Fabrication and characterization of blind‐hole NAA membranes produced by the two‐step anodization process. (a) Schematic with a definition of the main geometric features of the nanopore architecture in blind‐hole NAA membranes fabricated by the two‐step anodization process (i. e., nanopore diameter *D_P_*, interpore distance *D_Int_*, nanopore length *L_P_*, and barrier oxide layer thickness *τ_BOL_*). (b) Representative FEG‐SEM images of a blind‐hole NAA membrane showing the top, cross‐sectional and bottom views with its characteristic geometric features (scale bars: (i) 250 nm, (ii) 10 μm, (iii) 250 nm, and (iv) 250 nm).

To establish the general application of the model we also analyze a different system, the rectifying and dynamical properties of blind‐hole nanoporous anodic alumina (NAA) membranes as those shown in Figure 2.[^9^, ^23^, ^62^, ^63^, ^64^, ^65^] The membranes feature honeycomb‐like arrays of cylindrical, self‐organized nanopores characteristic of the two‐step anodization process with well‐defined geometric features. The nanopores of self‐organized blind‐hole NAA membranes have a homogeneous nanopore diameter of *D_P_* =45±5 nm and an interpore distance of *D_Int_* =108±10 nm (Figure 2b(i)). The bottom tip of each nanopore is closed by a hemispherical layer of anodic aluminum oxide (i. e., BOL), the thickness of which is measured to be *τ_BOL_* =45±2 nm (Figure 2b(ii) and b(iii)). The nanopores have a nominal length of 45±1 μm from top to bottom of the anodic membrane (Figure 2b(iv)). Blind‐hole NAA membranes were electrochemically characterized under symmetric electrolyte conditions (i. e., fixed concentration of KCl at 0.1 M and pH 6), with electrolyte of the open side of the pores grounded and the potential applied to the electrolyte of the bottom side.

**Figure 3:**
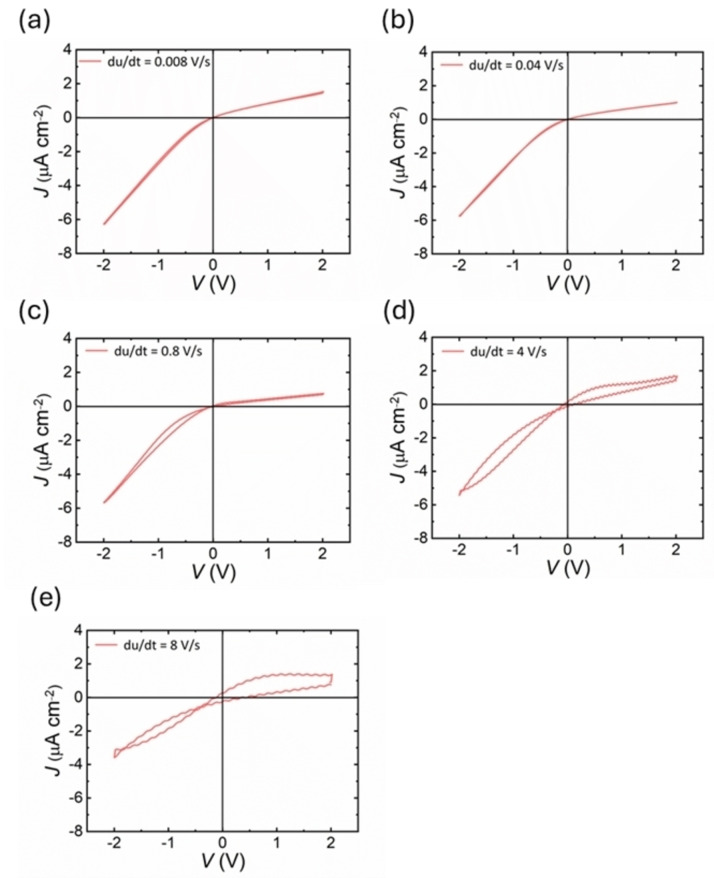
Analysis of effect of frequency in the input cycle voltammetry on the hysteresis response of blind‐hole NAA membranes by cyclic voltammetry using symmetric KCl electrolyte at a concentration of 0.1 M and pH 6. Range of frequencies used: ($f_{\Omega }=\;1,\;0.75,\;0.5,\;0.1,\;0.01,\;0.005,\;0.001$ Hz).

Using a biopotentiostat, the J–V characteristics were analyzed by applying a potential from anode (inner side of BOL) to cathode/ground (outer side of BOL) between two single junction silver chloride (Ag/AgCl, saturated KCl) reference electrodes. Hysteretic J–V profiles were acquired via cyclic voltammetry with triangular wave inputs at voltage amplitude of 2 V and varying frequency (0.001–1 Hz). In Figure 3 is shown the dynamical hysteresis response of the system at different voltage sweep rates. At very low frequencies, Figure 3a and b, rectification properties can be observed perfectly. The current is higher in the negative voltage window than in the positive due to the space charge density distributed across the layer of anodic oxide closing the bottom tips of the nanopores, and there is barely any relative hysteresis. On the other hand, as the frequency of the cycle voltammetry is increased, Figure 3c and d, the relative hysteresis effect increases as well, and inductive hysteresis is observed at negative voltages. For the fast measurement in Figure 3e the current at negative voltages does not reach the high steady state value but remains at a lower level, and the staircase‐like curves are associated with the ion transport behaviour across the BOL of NAA at higher scan rates.

The rectifying behavior of blind‐hole NAA (Figure 3) is attributed to the space charge density gradient present across BOL, rather than the aspect ratio of NAA. The volume of BOL consists of aluminum and oxygen vacancies that are generated from the migration of electrolytic ions during the growth of NAA. These vacancies are distributed in such a way that the concentration of aluminum vacancies at BOL near metal/oxide interface is higher than that of oxide/electrolyte. This generates a net space charge density gradient along the thickness of BOL, with net negative and positive space charge density at metal/oxide and oxide/electrolyte interfaces, respectively. The presence of space charge gradient affects the ion migration across the BOL of blind‐hole NAA membranes, which results in characteristic ionic current rectification. Therefore, the thickness of BOL has a stronger influence on the rectifying efficiency of blind‐hole NAA membranes than that of aspect ratio of NAA.[^6^, ^7^]

## 3 Model for Nanochannel Current‐Voltage Response

The model is inspired in HH neuron model,[^50^, ^51^, ^52^, ^53^, ^54^] related to the standard approach to a memristor.[^66^, ^67^, ^68^, ^69^] The form of the exponential relaxation times is adopted from previously reported memristor models.[^46^, ^69^, ^70^, ^71^]

The model consists of the set of equations:

(4) $$V_{app}=R_{s}I_{tot}+u$$

(5) $$I_{tot}=\left[g_{L}+\left(g_{H}-g_{L}\right)\;x\right]u+C_{m}\frac{du}{dt}$$

(6) $$\frac{dx}{dt}=\frac{\left(1-x\right)}{\tau_{s}\left(u\right)}-\frac{x}{\tau_{r}\left(u\right)}$$

(7)

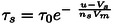



(8)

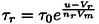

(9) $$\tau_{t}={\left(\frac{1}{\tau_{s}}+\frac{1}{\tau_{r}}\right)}^{-1}$$

Here $I_{tot}$ is the electrical current and $V_{app}$ the applied voltage. $R_{s}$ is the series resistance of the electrochemical setup and u the voltage across the nanopore. $g_{L}$ and $g_{H}$ are the conductances of the low and high conductance state, respectively. By Eq. (5) the conductance depends on the state of the blocking variable x . The state variable x indicates the charging of the nanopore from a low charge state at negative voltage (x=0 ) to the high conductivity (at x=1 ). $C_{m}$ is the membrane capacitance. $\tau_{s}$ and $\tau_{r}$ are the nonlinear relaxation (characteristic) times for set and reset cycles. The form of the relaxation time[^69^, ^70^] is an important factor determining hysteresis, as the nonequilibrium current will decay to the steady state value when the relaxation time becomes short. Hence $V_{s}$ and $V_{r}$ are the threshold voltage of the set and reset respectively. $V_{m}$ is a steepness parameter.

**Figure 4:**
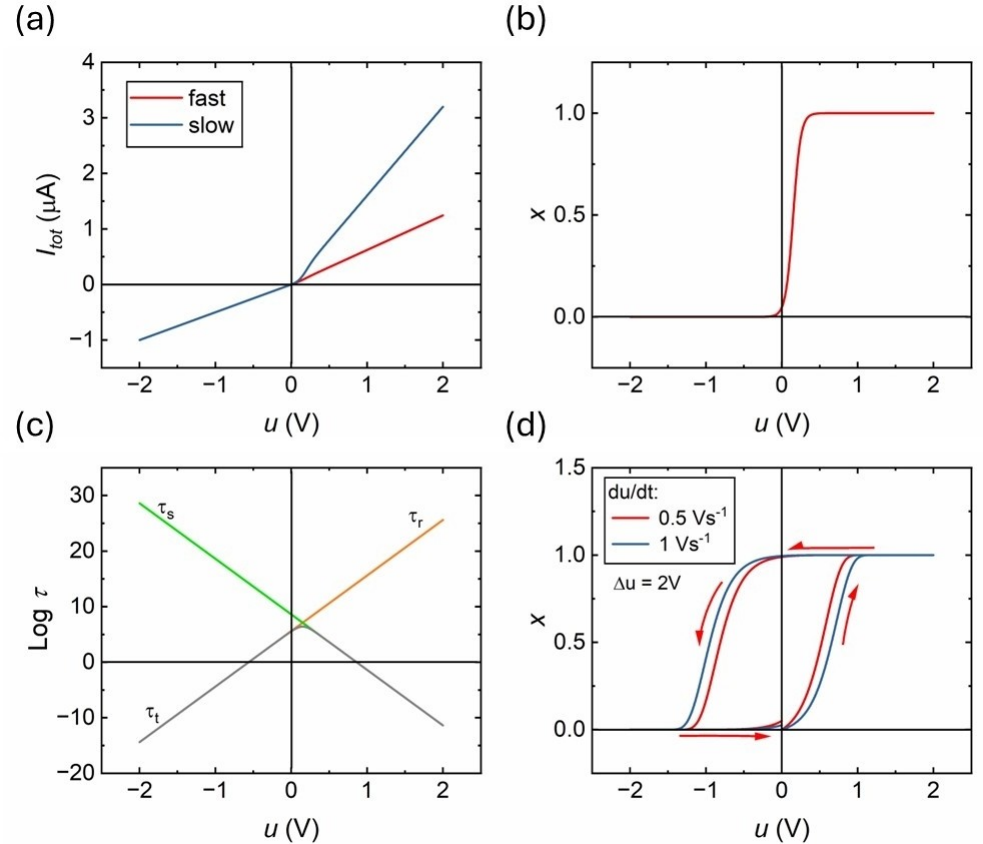
Representation of model functions, with respect to the voltage in the nanopore. (a) The current‐voltage curve at steady state and under a fast voltage sweep. (b) The state variable x at steady state. (c) Representation of the set time constant $\tau_{s}$ (green) and reset time constant $\tau_{r}$ (orange) associated with the state variable x . Grey line is the evolution of the total time constant $\tau_{t}$ . (d) Evolution of the state variable x at different voltage sweeps. Parameters:

In the steady state we obtain the expressions shown in Figure 4

(10) $$I_{tot}=\left[g_{L}+\left(g_{H}-g_{L}\right)x_{ss}\right]u_{dc}$$

(11) $$x_{ss}=\frac{1}{1+e^{\frac{-(u-V_{T)}}{n_{t}V_{m}}\;}}$$

The function $x_{ss}$ is activated at the voltage $V_{T}$ , defined by the following equations:

(12) $$n_{t}={\left(\frac{1}{n_{s}}+\frac{1}{n_{r}}\right)}^{-1}$$

(13) $$V_{t}=\left(\frac{V_{s}}{n_{s}}+\frac{V_{r}}{n_{r}}\right)n_{t}$$

The model makes use of a sigmoidal activation function (Equation (11)). This formulation corresponds to Boltzmann open channel probability distribution, which indicates the fraction of time that the conductivity channel is open according to the applied voltage. Equations (10 and 11) describe well the rectification property, since $x_{ss}\approx 1$ only at $V>V_{T}$ , Figure 4a.

The hysteresis in current‐voltage curves is well established in nanopore rectifiers, as we have shown in Figure 1 and 3. The hysteresis effect is caused by the delay of x with respect to the equilibrium value $x_{ss}$ , determined by the dynamical Equation (3). Obtaining $x_{ss}\approx 1$ requires a time longer than $\tau_{s}$ , otherwise the branch with high conductance $g_{H}$ in Equation  (5) is not formed, Figure 4a (red line), and the current will be restricted to the “fast” value:

(14) $$I_{fast}=\frac{V_{app}}{R_{s}+1/g_{L}}$$

**Figure 5:**
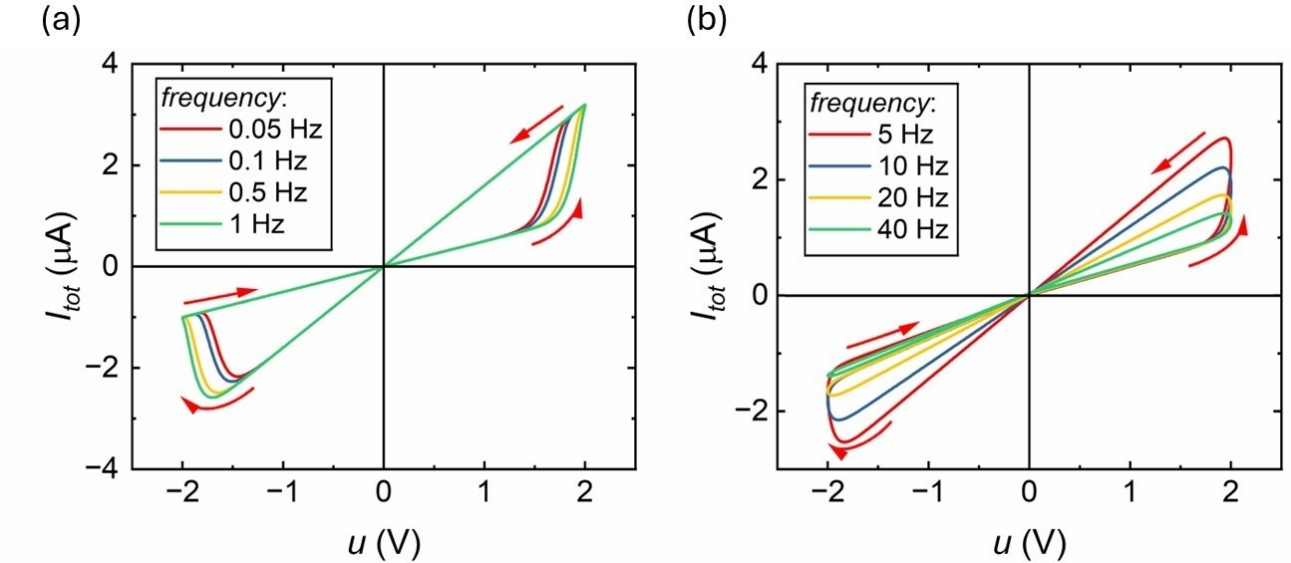
Representation of current loop with respect to the voltage in the nanopore at different pulse frequencies, same parameters as Figure 4. (a) The current‐voltage curve at low voltage frequencies (b) The current‐voltage curve at high voltage frequencies.

Here we show in Figure 5b the properties of hysteresis of the model defined by the Equations (5 and 6) and a cycling frequency $f_{\Omega }=\Omega_{s}/2\pi$ .. At low frequencies, the current‐voltage nearly follows the equilibrium curve of Figure 4a. But, as the frequency is increased, the opening that appears in the curve enhances, so that the hysteresis effect is increased. The current difference existing between the equilibrium curve and the hysteresis loop is inductive at positive voltages to the crossing point. After the equilibrium point, at negative voltage side, the evolution of the current with respect to the voltage is capacitive, as indicated by the arrows. Both inductive and capacitive properties arise from the delayed current mechanism.^[71]^

**Figure 6:**
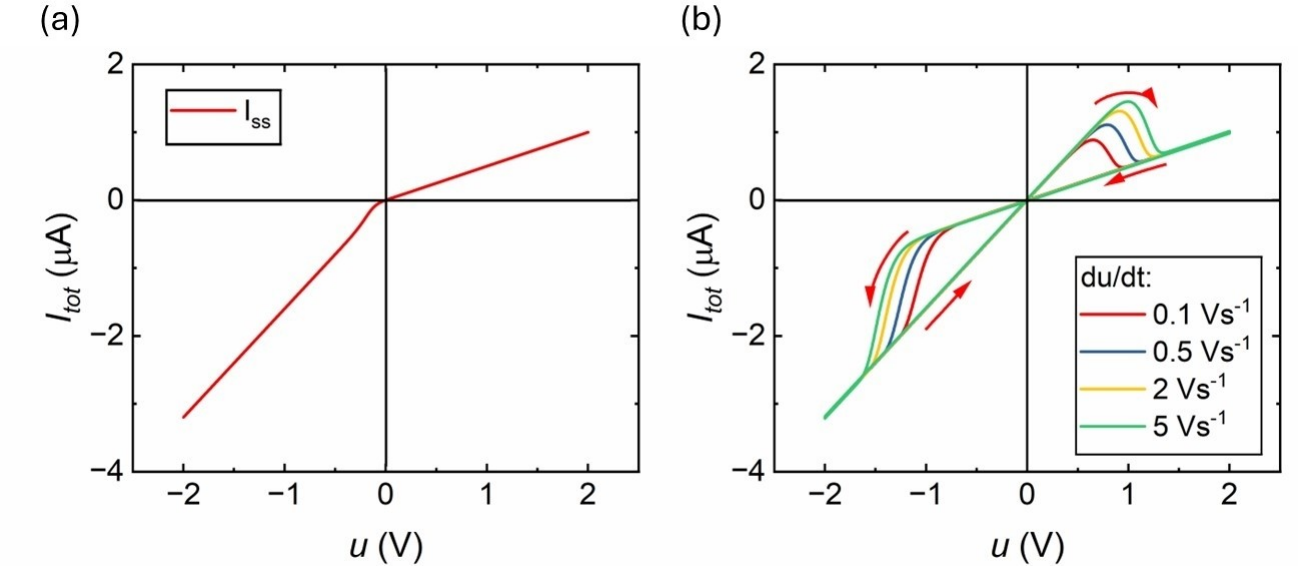
Representation of the variables of the model with respect to the voltage Applied voltage amplitude ΔV=2V . (a) Current in steady state which shows the rectification phenomena of the model. (b) Current‐voltage curve at different sweep voltage. Parameters: C_m_=6 ⋅ 10^−9^ F, R_S_=250000Ω, g_H_=1.4 ⋅ 10^−^6 Ω^−1^, g_L_=0.6 ⋅ 10^−6^ Ω^−1^, C_P_=1 ⋅ 10^−9^ F, V_m_=0.1, V_r_=0.4 V, V_S_=−0.7 V, n_r_=4, n_s_=4.

Modelling of blind‐hole nanoporous alumina of Figure 3 is shown in Figure 6. In this case the higher current branch is obtained at negative potentials. In Figure 6b at slow voltage scan rates, there is less hysteresis effect. Contrary, as the frequency of the cycle voltammetry is increased, the effect of hysteresis is also enhanced. Please note that no attempt was made to fit parameters of the data, which requires additional experimental methods as impedance spectroscopy. The simulation curves show that the model reproduces the dominant kinetic features of the hysteresis effects.

## 4 Synapse Properties of the Dynamic Response

Many works have presented results on the synapsis replication of nanochannels.[^24^, ^36^, ^37^, ^38^, ^39^, ^40^, ^41^, ^42^, ^43^] Here we investigate the characteristic behaviour of the polymer membrane of Figure 1 in order to provide an explanation of the dynamical behaviours using the model of Section 3.

**Figure 7:**
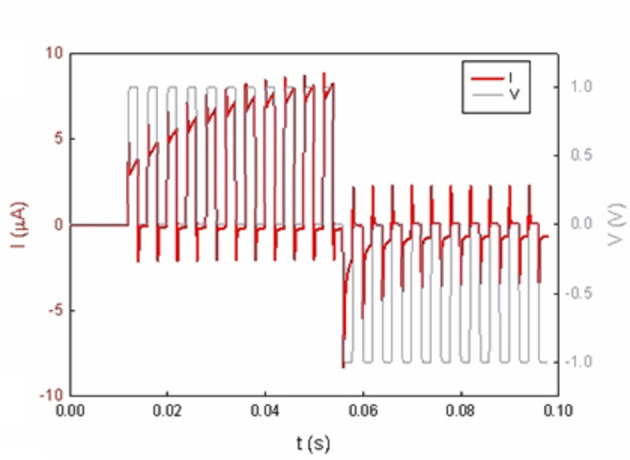
Current response of sample of Figure 1 to a train of 2 ms pulses showing a gradual inductive potentiation followed by a train of negative voltage pulses which reset the device to its original high resistive state.

Figure 7 shows that the response of the synaptic device to a train of positive potentiation voltage pulses is an increase of conductance, and the application of negative depressing voltage pulses is a decrease of conductance. The repeated application of positive stimuli decreases the resistance gradually and the resistive state of the device can be regulated to the desired value. The nanopore device is capable to emulate the biological synapses by a local modulation determined by the amplitude and height of the voltage stimulus.

**Figure 8:**
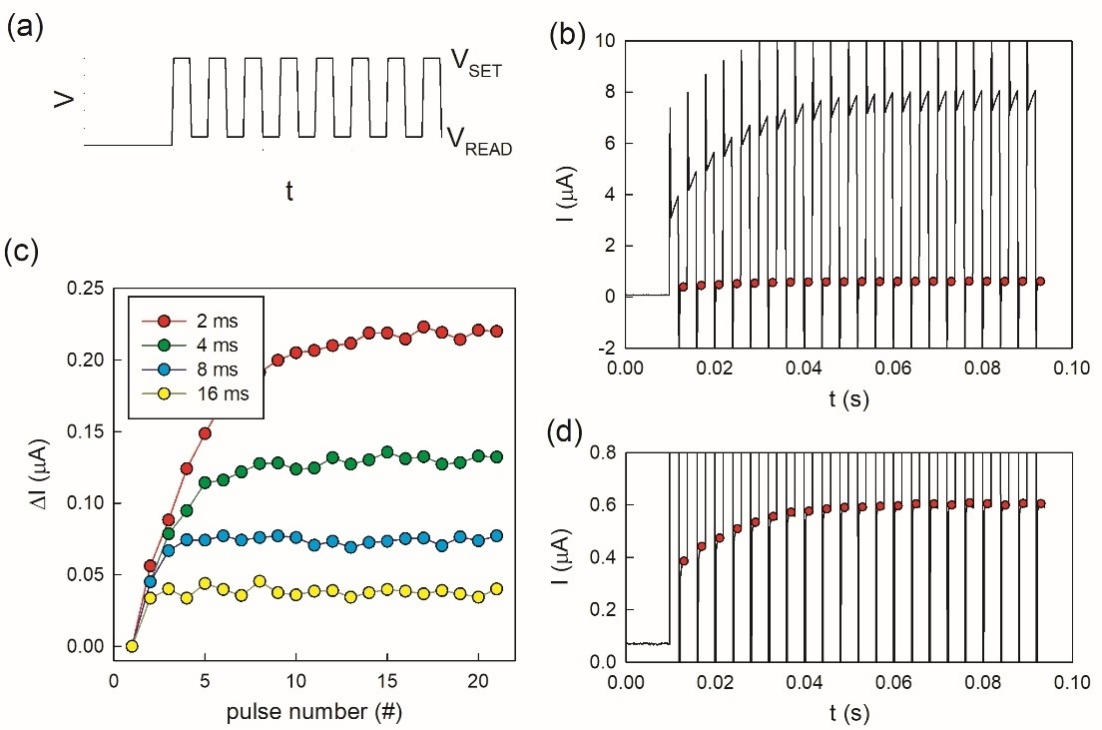
Average current increase of reading current value after a train of voltage steps of 1 V to the polymer membrane of Figure 1, with different reading times between set voltage steps with the read voltage of 0.1 V. (a) shows the schematic voltage stimulation given to the samples (b) shows the complete current response to a train of 20 voltage steps of 2 ms separated by 2 ms reading steps and (c) represents the different current increase for different reading time values. (d) is an expanded view of (b) and shows the detailed current response at the reading voltage scale.

In order to control the resistance state of the device after stimulation, it is needed to read a current value after the applied stimuli. Therefore, the required protocol to check the change in conductivity is to apply a read voltage after each voltage pulse. This is represented schematically in Figure 8a, where every pulse is followed by a read voltage period. When applying a train of voltage pulses, Figure 8b, the time lapse between the pulses affects the stimulation and the change in conductivity, as shown in the read values in Figure 8d. The more frequent the pulses are, the higher the stimulation is. Figure 8c shows the stabilized current value that is obtained by trains of equal voltage pulses which have different reading times between pulses. These results show that the device conductance can be incrementally adjusted by tuning the duration and sequence of the applied programming voltage, reaching different resistance states.

**Figure 9:**
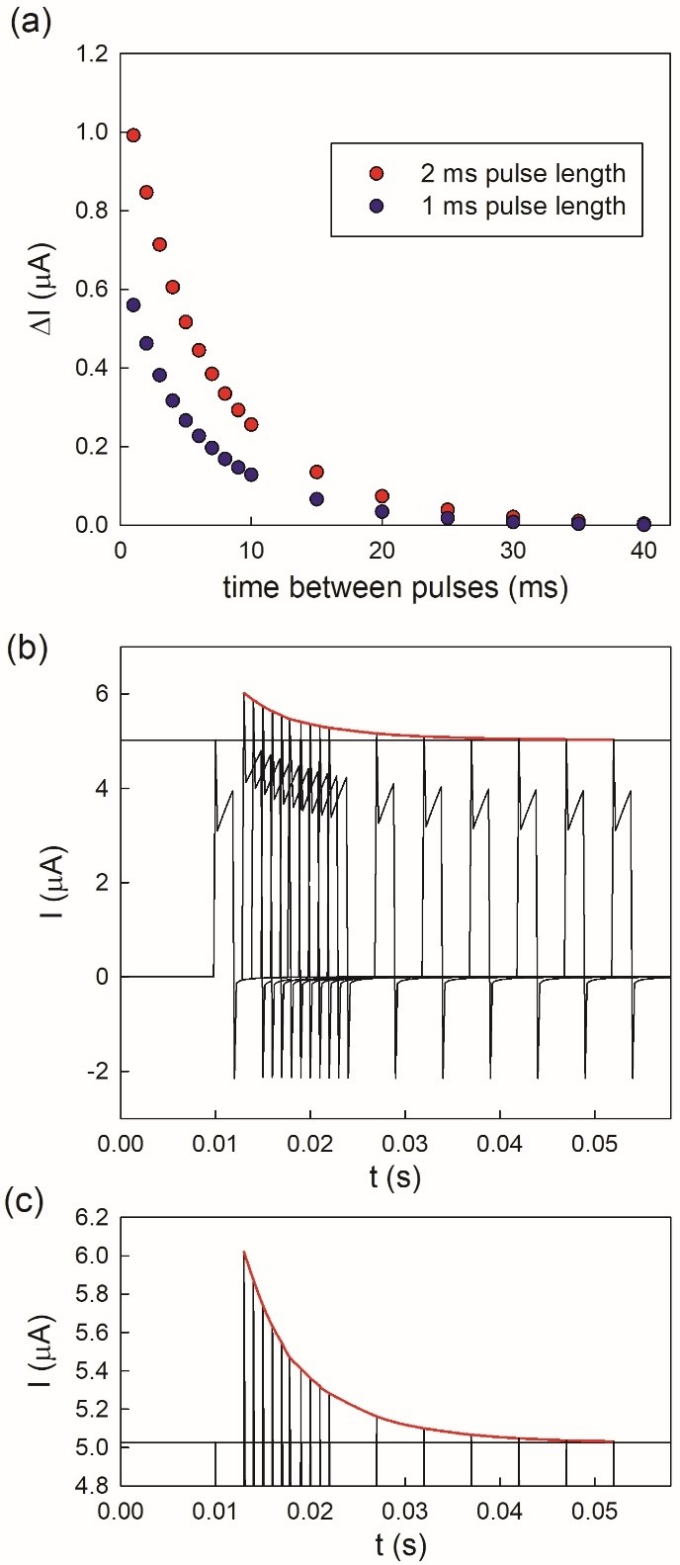
Paired‐Pulse Facilitation (PPF) analysis of sample #1. A pair of voltage steps is applied to the nanoporous sample and the response of the second step is compared to the first one, this protocol is carried out at different time separations between the two steps and, in this case, two different time lengths (1 ms and 2 ms) of the voltage steps. (a) represents the decay of the current difference when the time between pulses is increased, up to 40 s, where the initial step is totally recovered. (b) shows the responses of the pair of steps of 2 ms length with different separation and (c) is the detail of the difference between the first step response and the following steps. The flat black line represents the first step in comparison with the evolution of the following steps in red.

PPF consists of an enhancement in the amplitude of the second of two rapidly evoked excitatory postsynaptic potentials. Figure 9 shows the PPF behaviour of the rectifying nanopore. It is observed that the second pulse when it is immediately close to the first one increases notably the current response, and it diminishes as the separation between pulses increases. We can see that the second pulse is equal to the first pulse when the separation between pulses reaches 40 ms, and the initial resistance state has been reestablished.

## 5 Modelling the Synaptic Response

The potentiation/depression phenomena observed in Figure 8 correspond to current transient responses of the synaptic nanopore. We now apply the general model of Equations (4–9) to provide an interpretation of the observed potentiation phenomena, based on the detailed method of analysis of transient pulses presented recently.^[46]^

**Figure 10:**
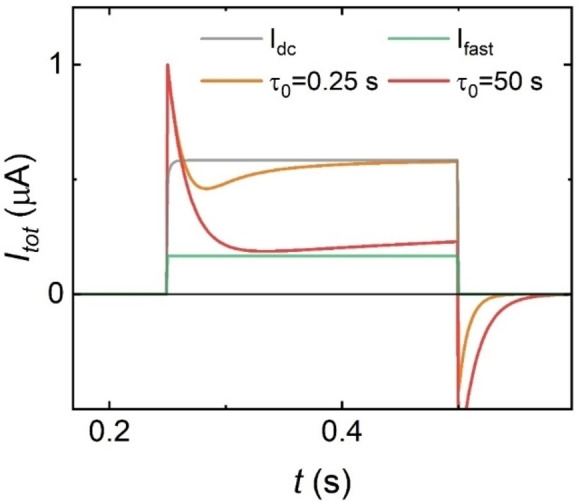
Model calculation of the current response to a square voltage pulse from V=0 at $t_{0}=1$ of duration Δt=0.25s and size ΔV=2V .Orange line for a short and red line for a long kinetic time $\tau_{0}$ of the inductive process. The green current line is that when x=0 and the grey line is the final steady state current. Parameters: C_m_=1 ⋅ 10^−8^ F, R_S_=2 MΩ, g_H_=0.7 ⋅ 10^−^6 Ω^−1^, g_L_=0.1 ⋅ 10^−6^Ω^−1^, V_m_=0.1, V_r_=−0.7 V, V_S_=0.4 V, n_r_=4, n_s_=4.

In Figure 10 we show the current response to a square voltage pulse of duration Δt and size ΔV . An initial peak is due to the localization of the voltage in the series resistance at the first instant, and subsequent charging of the capacitor. The next evolution depends dramatically on the kinetics of the slow inductive process. For large $\tau_{0}>\Delta t$ (red line) the slow current cannot be onset, and the transient finishes close to the fast current (green) value of Equation (14). In this scenario, the inductor is barely present in the response and more pulses (time) are required to be completely activated. The current will reach the steady state value (green line) after several voltage pulses.

On the other hand, when $\tau_{0}\leq \Delta t$ (red line) the slow current is developed and the current increases with time after the initial capacitive decline. This increase of current associated to the inductor effect is the core of the synaptic function. The current can reach the final steady state value, or not, depending on the interplay of the $\tau_{0},\;\Delta t$ values. At values of initial time constants$\;\tau_{0}$ smaller or equal to the period of the voltage pulse Δt , the inductor is activated completely, and the current can reach the steady state value in initial pulse. In the experimental results of Figure 7 it is observed that the current rise is interrupted by the cessation of the voltage stimulus, and continues in the next step, so that the current increases gradually in the potentiation phenomenon. Another feature explained by the model is the negative peak observed after each voltage square cycle in Figure 7 and 9. This is due to the formation of a negative current to remove the charge in the capacitor.^[56]^

**Figure 11:**
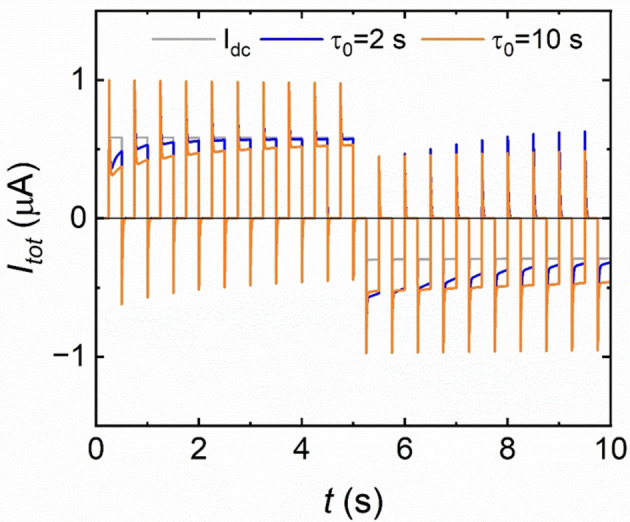
Model current response to a square positive and negative voltage pulses from V=-2 to V=2 with a pulse period =0.5s . Orange current is the response under kinetic time $\tau_{0}=10$ s and blue current is the response to a shorter time constant $\tau_{0}=2$ s. The grey line is the final steady state current related to the high conductivity state. Parameters: C_m_=1 ⋅ 10^−8^ F, R_S_=2 MΩ, g_H_=0.7 ⋅ 10^−6^Ω^−1^, g_L_=0.1 ⋅ 10^−^6 Ω^−1^, V_m_=0.1, V_r_=−0.7 V, V_S_=0.4 V, n_r_=4, n_s_=4.

According to these kinetic properties, the model presented in Section 3 can reproduce the typical synaptic response of a polymer membrane of Figure 7,^[43]^ as shown in Figure 11. Under the application of positive voltage pulses, an increase of the conductance with the time is observed in the orange line corresponding to $\tau_{0}=$ 10 s, perfectly reproducing potentiation phenomena. On the other hand, under negative applied voltage pulses, a depression of the conductance is reported as a gradually decrement of the current. In Figure 11 is shown that with a short time constant (blue current), the current reaches the steady state value (grey line) before than with the longer time constant (orange current).

**Figure 12:**
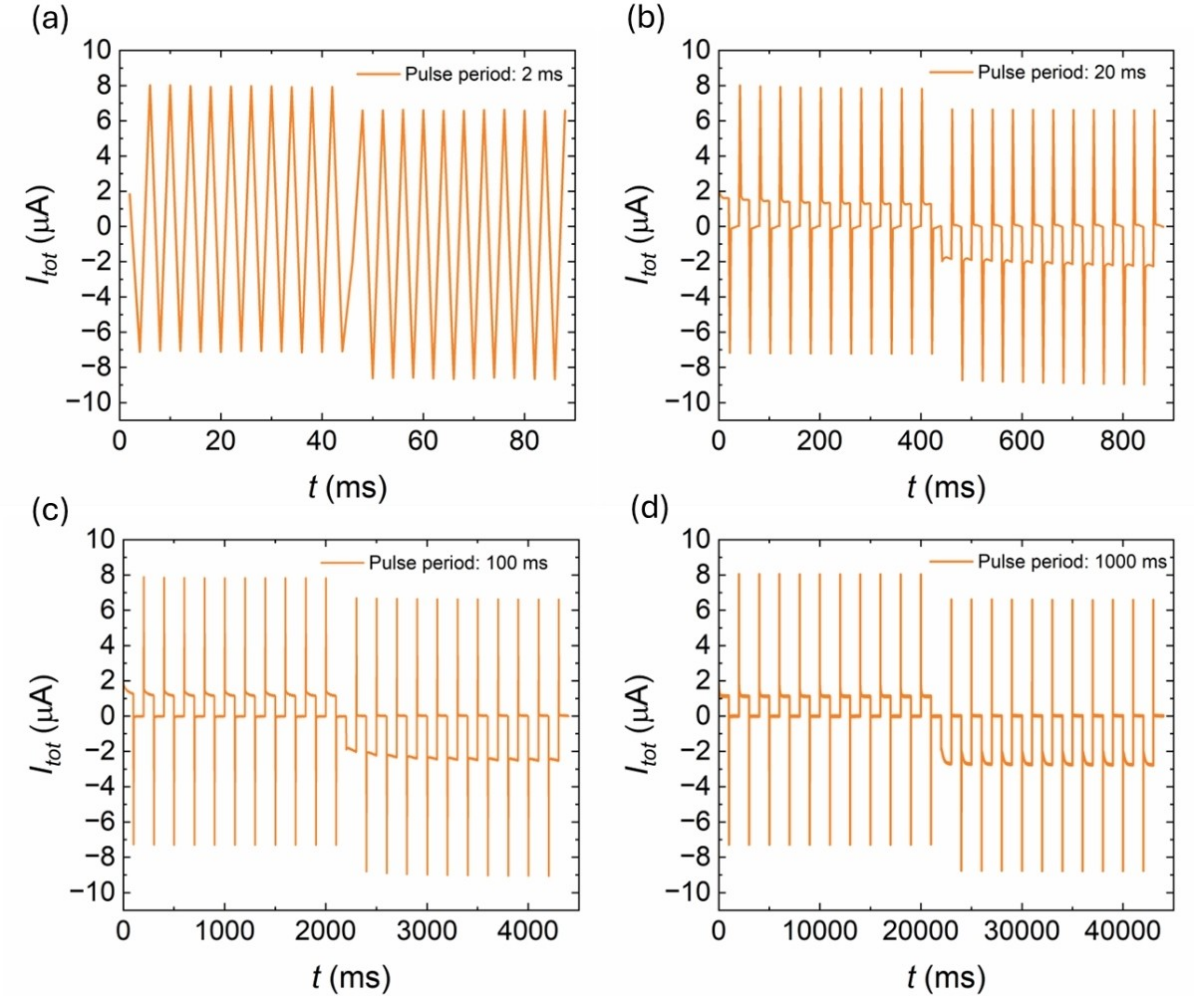
Experimental effect of the pulse time activation in the transient response of blind‐hole NAA membranes after applying positive and negative train voltage pulses of different period. Voltage amplitude ΔV=[-22]V and different pulse period T=[220,100,1000]ms . (a) Time response of the current at train voltage of period equal to 2 ms. (b) Time response at train voltage of period 20 ms. (c) Time response at train voltage of period 100 ms. (d) Time response at train voltage of period 1000 ms.

We now investigate the transient current response of blind‐hole NAA membranes under a train of voltage pulses of different duration, as shown in Figure 12. Due to the polarity of the system shown in Figure 3, potentiation effect is only observed at negative voltage pulses. At very small pulse period (Figure 12a) the response is totally capacitive and there is not an effect of potentiation. This is because the inductive branch is not activated yet. At more intermediate pulse period (Figure 12b and c), the current response shows both types of behavior. First an initial drop occurs due to the fast capacitance that is followed by a progressive increase (potentiation effect) until it reaches the steady state current, Figure 12c. At very long pulse period (Figure 12d), the response to negative voltage pulses shows the increase of current inside each cycle. The time constant associated with the inductor is shorter than the pulse period of the voltage wave, in other words, the pulse period is long enough to let the inductor be fully activated.

**Figure 13:**
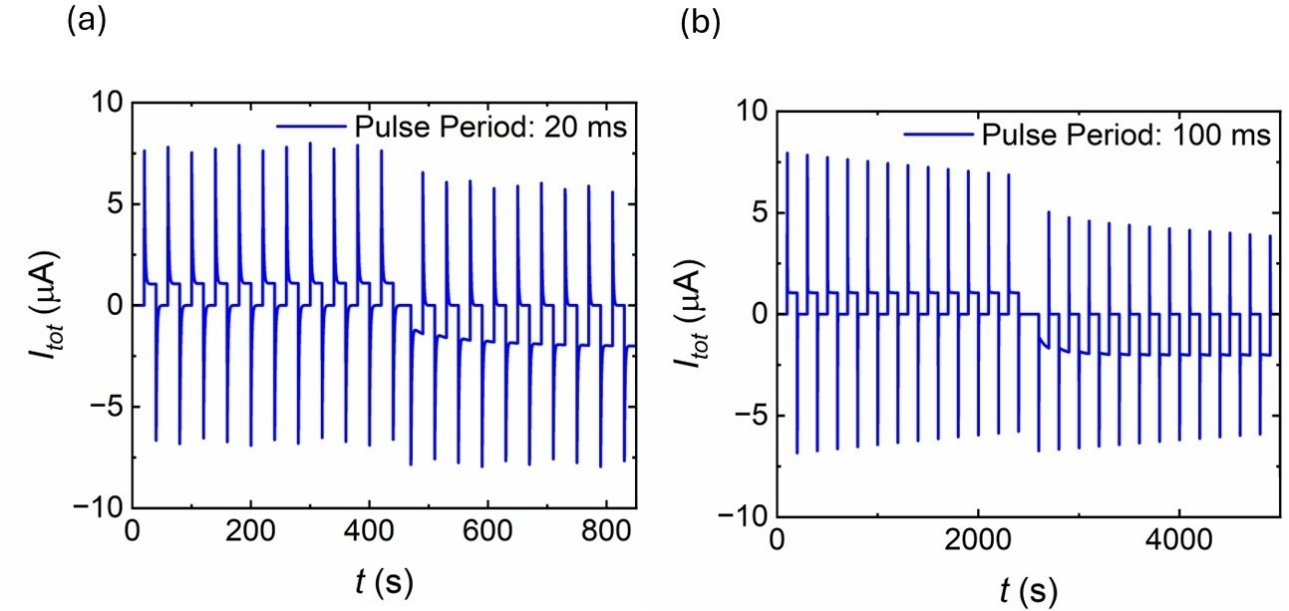
Model representation of the influence exerted by the pulse period of the applied voltage $V_{app}$ on the current time response. Initial positive voltage pulses from V=0V to V=2V followed by negative voltage pulses from V=0V to V=-2V . (a) Capacitive current response at a voltage pulse equal to 20 ms. (b) Inductive current response at a voltage pulse of 100 ms: C_m_=6 ⋅ 10^−9^ F, R_S_=250000 Ω, g_H_=1.4 ⋅ 10^−6^Ω^−1^, g_L_=0.6 ⋅ 10^−6^ Ω^−1^, C_P_=1 ⋅ 10^−9^ F, V_m_=0.1, V_r_=0.4 V, V_S_=−0.7 V, n_r_=4, n_s_=4, τ_0_=5 s.

The model described in Section 3 can indeed reproduce synaptic properties of blind hole nanopores observed in Figure 12. The results of the calculations are shown in Figure 13. As expected, at shorter pulse period, Figure 13a, the current response is capacitive at positive pulses since the inductive branch of the model is not activated. This is due to the state variable x not being triggered. At negative voltage pulses, the state variable x is initiated resulting in the activation of the inductive branch. As a result, potentiation effect appears as a gradually increase of the current. If the period of the pulses is increased as in Figure 13b, at negative voltage pulses potentiation effect can be observed as well.

## 6 Conclusions

The rectifying and hysteresis properties of conical nanopores supply the necessary conductance adaptations to program the device by adjusting the synaptic weight according to the duration and strength of the received stimuli. We have derived a kinetic model that allows the quantitative analysis of current‐voltage curves, hysteresis effects, and the response to a train of voltage pulses that determines the current enhancement and depression in potentiation effects. By applying the model to the experimental behaviour of different types of fluidic nanopores, we showed that the model mechanism is fairly general so that the approach holds promise for improving the performance of fluidic networks for brain‐like computation applications.

## Conflict of Interests

The authors declare no conflict of interest.

7

## Data Availability

The data presented here can be accessed at https://doi.org/10.5281/zenodo.13249856 (Zenodo) under the license CC‐BY‐4.0 (Creative Commons Attribution‐ShareAlike 4.0 International).
